# Drug Recommendation System for Diabetes Using a Collaborative Filtering and Clustering Approach: Development and Performance Evaluation

**DOI:** 10.2196/37233

**Published:** 2022-07-15

**Authors:** Luis Fernando Granda Morales, Priscila Valdiviezo-Diaz, Ruth Reátegui, Luis Barba-Guaman

**Affiliations:** 1 Departamento de Ciencias de la Computación y Electrónica Universidad Técnica Particular de Loja Loja Ecuador

**Keywords:** clustering, collaborative filtering, diabetes, recommender system, recommend, drug, chronic disease, patient information, data mining, machine learning

## Abstract

**Background:**

Diabetes is a public health problem worldwide. Although diabetes is a chronic and incurable disease, measures and treatments can be taken to control it and keep the patient stable. Diabetes has been the subject of extensive research, ranging from disease prevention to the use of technologies for its diagnosis and control. Health institutions obtain information required for the diagnosis of diabetes through various tests, and appropriate treatment is provided according to the diagnosis. These institutions have databases with large volumes of information that can be analyzed and used in different applications such as pattern discovery and outcome prediction, which can help health personnel in making decisions about treatments or determining the appropriate prescriptions for diabetes management.

**Objective:**

The aim of this study was to develop a drug recommendation system for patients with diabetes based on collaborative filtering and clustering techniques as a complement to the treatments given by the treating doctor.

**Methods:**

The data set used contains information from patients with diabetes available in the University of California Irvine Machine Learning Repository. Data mining techniques were applied for processing and analysis of the data set. Unsupervised learning techniques were used for dimensionality reduction and patient clustering. Drug predictions were obtained with a user-based collaborative filtering approach, which enabled creating a patient profile that can be compared with the profiles of other patients with similar characteristics. Finally, recommendations were made considering the identified patient groups. The performance of the system was evaluated using metrics to assess the quality of the groups and the quality of the predictions and recommendations.

**Results:**

Principal component analysis to reduce the dimensionality of the data showed that eight components best explained the variability of the data. We identified six groups of patients using the clustering algorithm, which were evenly distributed. These groups were identified based on the available information of patients with diabetes, and then the variation between groups was examined to predict a suitable medication for a target patient. The recommender system achieved good results in the quality of predictions with a mean squared error metric of 0.51 and accuracy in the quality of recommendations of 0.61, which is acceptable.

**Conclusions:**

This work presents a recommendation system that suggests medications according to drug information and the characteristics of patients with diabetes. Some aspects related to this disease were analyzed based on the data set used from patients with diabetes. The experimental results with clustering and prediction techniques were found to be acceptable for the recommendation process. This system can provide a novel perspective for health institutions that require technologies to support health care personnel in the management of diabetes treatment and control.

## Introduction

### Background

Owing to the large amount of information available in health institution databases, including medical treatments, diagnostic tests, clinical histories, and drug characteristics, there is a need to implement recommender systems (RSs) that support medical staff in activities related to health control and management. The main concept of an RS is to suggest items that are particularly suitable for the user based on their profile or historical preferences. In the context of health, these items can be drugs, medical treatments, health videos, and patients sharing the same disease. An RS employs data sources to learn about user preferences through machine-learning algorithms and information-filtering techniques such as content-based, collaborative filtering–based, demographic, and hybrid approaches [1].

Currently, more than 80% of internet users seek health information through various platforms, including social networks, a figure that, according to De Choudhur et al [2], continues to grow. Through the internet, users can identify patients with the same disease, the possible causes of their ailments, find procedures to alleviate a particular disease, learn new healthy habits, and find general health information [3-5].

In the case of chronic diseases such as diabetes, the prescription of multiple drugs is common; thus, RSs can support the intervention of the treating doctor in determining which drugs to prescribe to a particular patient. Considering the current health status of a patient, their clinical history, medications prescribed in previous periods, specific symptoms, and other characteristics, the system can search for patients with similar parameters in the database and suggest drugs that have been more successful in these cases, which could be recommended to the target patient.

Some research has been performed on RSs in the health area. For example, Zhang et al [6] proposed the iDoctor system to provide users with personalized medical recommendations. This system explores users’ emotions and preferences about doctors through their ratings and reviews. Gujar et al [7] proposed data-mining techniques for the prediction of a disease and to make a recommendation for specialists about the predicted disease. Kuanr et al [8] proposed an RS for cervical cancer in which predictive models showed high accuracy. Poornima [9] presented a daily nutrition RS for women taking into consideration physical data, preferences, and personal information to combat diseases such as malnutrition, obesity, and cardiovascular diseases.

Other research has focused primarily on recommending doctors and hospitals that are best suited to a specific patient profile [10], medication recommendations [11], treatment recommendations for patients over time [12], videos about health [13], and even customized meal plans [14].

Recently, several studies related to the use of RSs in diabetes have emerged, including some exploratory analyses on the disease, predictions on diet plans to combat obesity and diabetes [14], and physical activity and diet plan RSs to help prevent chronic diseases [15].

Clustering is one of the most widely used machine-learning techniques in the field of health to identify patterns or groups of patients with similar characteristics [16,17]. Although the clustering technique has been the subject of research in the area of RSs, these systems have not yet been widely used in medicine. Moreover, technologies that enable only the analysis or prediction of diagnoses or diseases would not be sufficient to provide personalized care to the patient or to support health personnel in making decisions about which drugs to consider for certain diseases. Therefore, we here propose a drug RS for patients with diabetes based on clustering techniques as a complement to the treatments given by the treating doctor.

Both drug predictions and recommendations were evaluated using traditional metrics to measure the performance of the RS.

### Contribution

The aim of this study was to complement previous diabetes-related studies by first analyzing data related to patients with diabetes to obtain important information for the management of this disease, followed by identifying groups of patients who share similar characteristics, which could enable discovering patterns of interest that can support decision-making. Finally, an RS was developed that suggests medications for diabetes according to the patient’s historical information and the doses of the medications administered.

## Methods

### Data Set

The data set used was obtained from the University of California Irvine (UCI) Machine Learning Repository [18], which contains information on patients with diseases associated with diabetes [19]. The original data set includes more than 50 features representing patient outcomes from 130 US hospitals. This data set has more than 100,000 patient records, which refer to 10 years of health care records (from 1999 to 2008).

In summary, the information contained in the data set refers to: admissions of patients to the hospital; information on 24 medications administered to patients with diabetes; changes in the patients’ medication, and whether the dosage was increased, reduced, kept stable, or not administered; number of medications administered to the patient; time spent by the patient in the hospital, recorded in days; results of tests that were indicated to patients prior to and during their treatment; diagnosis; type of admission; specialty of the treating doctor; and patient data such as age, race, and gender.

### Exploratory Data Analysis

Exploratory analysis of the data set is a critical process in research to discover patterns, detect anomalies, test hypotheses, and test assumptions with the help of statistics and graphical representations. It is good practice to first understand the data to obtain as much information as possible.

**Figure 1:**
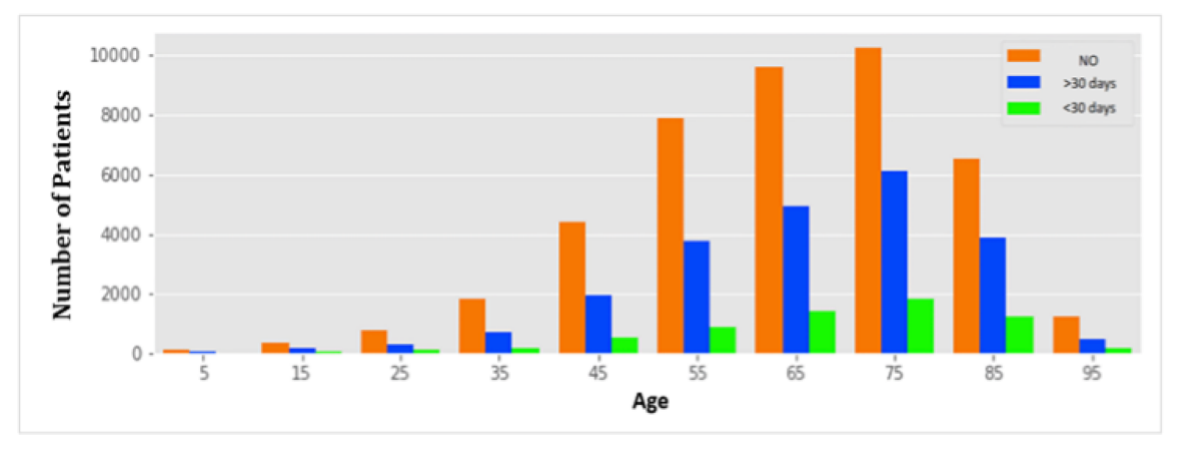
Distribution of patients according to age and readmission.

Toward this end, in the initial analysis of the patient data, we determined that most of the patients belong to the age range of 50 to 80 years, and we classified patients according to readmission status (ie, if the patient presents a case of readmission greater than or less than 30 days, and no readmission). Figure 1 shows the relationship between age and patient readmission, demonstrating very few cases of readmission for younger patients (under 40 years of age). In addition, women had a slightly higher readmission rate than men in cases of readmission longer than 30 days. Readmission showed a similar distribution for patients with and without medication prescribed for diabetes prior to hospital treatment. In addition, we determined that the majority of the patients were of the Caucasian race and did not have a glucose or hemoglobin A1C test.

### Data Preparation

#### Overview

Data preparation, also known as preprocessing, is a key task in the initial stages to ensure a correct analysis of the information available. Before applying the clustering techniques, the original data set was cleaned by removing all duplicated cases of patients and eliminating records of patients who had not been prescribed any medication. We then performed data transformation and variable selection.

#### Data Transformation

The data transformation process generally involves converting variables to another type of data and creating new variables. In our case, we converted categorical variables to binary variables, such as gender, maximum glucose serum level, and hemoglobin A1C test result.

New variables were created from the categories of the first diagnosis variable, which was coded according to the first three digits of the International Classification of Diseases-9 system: circulatory, diabetes, digestive, genitourinary, injuries, musculoskeletal, neoplasms, respiratory, and other. The categories of the race variable were created as new variables. Subsequently, the first diagnosis and race variables were removed from the data set. In addition, the age ranges were replaced by the mean of the ranges.

#### Variable Selection

Noninformative features in the data set were discarded due to a large number of missing values (50,000/100,000, 50.00%) or because some features were not relevant to classifying the data, such as patient identification, or if the feature is unbalanced (n=95,000, >95% of the data had the same value for a feature). In addition, we selected patients who had been prescribed at least two medications. Table 1 lists the discarded parameters (features) and the reasons for discarding them.

As a result, a final data set was obtained with 5177 unique patient records and 42 variables, which were categorized as patient characteristics and medications administered to patients. These final attributes are detailed in Table 2 and Table 3, respectively.

Drugs whose administration represented a very small percentage (500/100,000, 0.50%) or drugs that had not been administered to any patient (as was the case for examide and cytoglipton) were eliminated. Table 3 lists the drugs selected after data processing and the number and proportions of patients using the drug.

For each drug, we classified whether it was administered or if there was a change in the dose. We considered four values for this variable: “up” indicates that the dose was increased during the patient encounter, “down” indicates that the dose was decreased, “steady” indicates that the dose did not change, and “no” indicates that the drug was not administered.

As shown in Table 3, insulin was administered to more than 50% of the patients present in our data set, metformin was administered to slightly less than 20% of the patients, followed by glipizide and glyburide. The drugs that were administered to fewer patients were glyburide-metformin and nateglinide.

**Table 1:** Variables discarded from the data set.

Variable	Discard reason
encounter_id^a^	Irrelevant variable for clustering
patient_nbr^b^	Irrelevant variable for clustering
payer_code^c^	Irrelevant variable for clustering
Weight	Data missing for 97.00% (n=97,000) of the 100,000 samples
Medical specialty	Data missing for 53.00% (n=53,000) of the 100,000 samples
Clorpropamida	Only 86 patients use this drug
Acarbosa	Only 308 patients use this drug
Miglitol	Only 38 patients use this drug
Troglitazona	Only 3 patients use this drug
Examide	No patient uses this drug
Citoglipton	No patient uses this drug
Glipizide_metformin	Only 13 patients use this drug
Glimepirida_pioglitazona	Only 1 patient uses this drug
Metformin_rosiglitazone	Only 2 patients use this drug
Metformina_pioglitazona	Only 1 patient uses this drug
Acetohexamida	Only 1 patient uses this drug
Tolbutamide	Only 23 patients use this drug
Tolazamide	Only 39 patients use this drug

^a^encounter_id: Identification of a specific hospital visit or patient encounter.

^b^patient_nbr: patient ID number.

^c^payer_code: identifier corresponding to 23 distinct values of payment method (eg, Blue Cross*/*Blue Shield, Medicare, patient payment).

**Table 2:** Patient characteristics, description, and their corresponding values.

Variable	Description	Values
Gender	Patient gender (self-identified)	0, 1
Age	Patient age (years)	5, 15, 25, 35, 45,…95
admission_type_id	Identifier corresponding to 8 different types of admissions: emergencies, accidents, newborns, and others	1-8
discharge_disposition_id	Identifier of the discharge type (eg, discharged to home, psychiatric hospital, medical facility)	1-28
admission_source_id	Identifier of the admission source (eg, transfer from hospice, transfer from an ambulatory surgery center)	1-11, 13-14, 20, 22, 25
time_in_hospital	Number of days between admission and discharge	1-14
num_lab_procedures	Number of laboratory tests performed during the encounter	1-132
num_procedures	Number of procedures performed during the encounter	0-6
num_medications	Number of different drugs (generic names) administered during the encounter	1-81
number_outpatient	Number of outpatient visits	0-42
number_emergency	Number of emergency visits	0-76
number_inpatient	Number of inpatient visits	0-21
number_diagnoses	Number of diagnoses	1-16
max_glu_serum	Range of the result of the serum glucose level or if the test was not performed	0, 1
a1cresult	Range of the result of the hemoglobin A1C level or if the test was not performed	0, 1
change	If there is a change in medication	0, 1
diabetesmed	If the patient has been prescribed medication for diabetes	0, 1
readmitted	Days to inpatient readmission; these categories will be relabeled	0, 1, 2
African American, Asian, Caucasian, Hispanic, Other	Patient’s race	0, 1
circulatory	If the patient is admitted with circulatory system problems, the variable takes the value of 1	0, 1
diabetes	If the patient is admitted with diabetes-related problems, the variable takes the value of 1	0, 1
digestive	If the patient is admitted with digestive system problems, the variable takes the value of 1	0, 1
genitourinary	If the patient is admitted with genitourinary problems, the variable takes the value of 1	0, 1
injury	If the patient is admitted with injuries, the variable takes the value of 1	0, 1
musculoskeletal	If the patient is admitted with musculoskeletal problems, the variable takes the value of 1	0, 1
neoplasms	If the patient is admitted with neoplasms, the variable takes the value of 1	0, 1
respiratory	If the patient is admitted with respiratory system problems, the variable takes the value of 1	0, 1
other2	If the patient is admitted with other complications, the variable takes the value of 1	0, 1

**Table 3:** Final drugs used in the data set among 24 total drugs (N=100,000 patients).^a^

Drug	Patients, n (%)
Insulin	54,383 (53.44)
Metformin	19,988 (19.99)
Glipizide	12,686 (12.69)
Glyburide	10,650 (10.65)
Pioglitazone	7328 (7.33)
Rosiglitazone	6365 (6.37)
Glimepiride	5191 (5.19)
Repaglinide	1539 (1.54)
Glyburide-metformin	706 (0.71)
Nateglinide	703 (0.70)

^a^Some patients were administered more than one drug.

### Clustering-Based Recommendation

#### General Approach

The proposed RS is based on the collaborative filtering approach to represent the drugs prescribed to each patient according to the dose given. The clustering technique was applied to group patients with similar characteristics.

**Figure 2:**
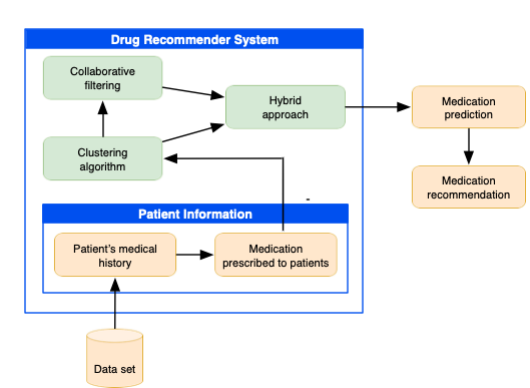
Schematic of the proposed recommendation approach.

Figure 2 shows a schematic of our proposed method, where the relationship between the elements of the system can be appreciated. The patient’s information is first obtained from the data set, such as clinical history, treatments, and the medication prescribed, and then the data are further processed for the application of clustering algorithms. The collaborative filtering technique is then applied to represent the patient’s explicit data (user-medication-dose). According to the group to which the patient belongs, the prediction of the medications is made. Finally, the recommendation is made considering the drugs with the highest prediction value.

#### Patient Grouping

To obtain the patient groups, we tested two clustering algorithms to determine the algorithm that provides the best result: the partitional K-means algorithm and the density-based spatial clustering of applications with noise (DBSCAN) algorithm. For this process, it was first necessary to normalize the data and reduce the dimensionality of the data set using principal component analysis (PCA). The optimal number of clusters was determined using the Silhouette coefficient. Patients sharing the same characteristics will be part of the same cluster.

The results of both clustering algorithms were compared using the value of the Silhouette coefficient obtained.

#### Drug Prediction

For calculation of the prediction performance, we randomly divided the data set into two parts: 80.00% (4142/5177) for training the algorithm and the remaining 20.00% (1035/5177) for testing.

The collaborative filtering approach requires users, items, and ratings; in our case, these elements were replaced by patients, drugs, and drug dosage, respectively, where a value of 1 means that the patient’s drug dosage was decreased and a value of 2 means that the drug dosage was increased. With these elements, we proceeded to the construction of the collaborative filtering matrix, as shown in Table 4.

The matrix was completed with the prediction of the drug dose value for the patients in each cluster, which was calculated by applying the cosine similarity measure for each of the clusters.

**Table 4:** Collaborative filtering matrix.

	Medication 1	Medication 2	Medication 3	Medication 4	Cluster
Patient 1	1	0	2	1	1
Patient 2	0	1	1	1	2
Patient 3	2	0	0	1	1
Patient 4	1	2	0	0	3
Patient 5	1	1	2	0	2

## Results

### Overall Clustering Results

First, PCA was used to reduce the dimensionality of the data set, resulting in 8 components explaining most of the variance of our data (ie, these components explained 62% of the total variance). We then applied the two clustering algorithms with the 8 principal components. Table 5 shows the best results of the experimentation with the K-means and DBSCAN algorithms.

In comparison with DBSCAN, the K-means algorithm had a higher Silhouette coefficient and a much lower number of clusters for the same data set. Moreover, K-means had a much faster execution time, which means that this algorithm presents lower computational complexity in terms of execution compared with that of DBSCAN. Therefore, the clustering results obtained with K-means were further considered for the calculation of prediction and recommendations. Analysis of the clusters was performed considering the clustering obtained with the K-means algorithm, which showed the best results.

Cluster 4 had the highest number of patients, followed by clusters 2 and 6. Cluster 3 had the smallest number of patients due to the different characteristics considered to group similar patients, such as the diagnosed diseases, race, main drugs administered, and most representative age range.

**Figure 3:**
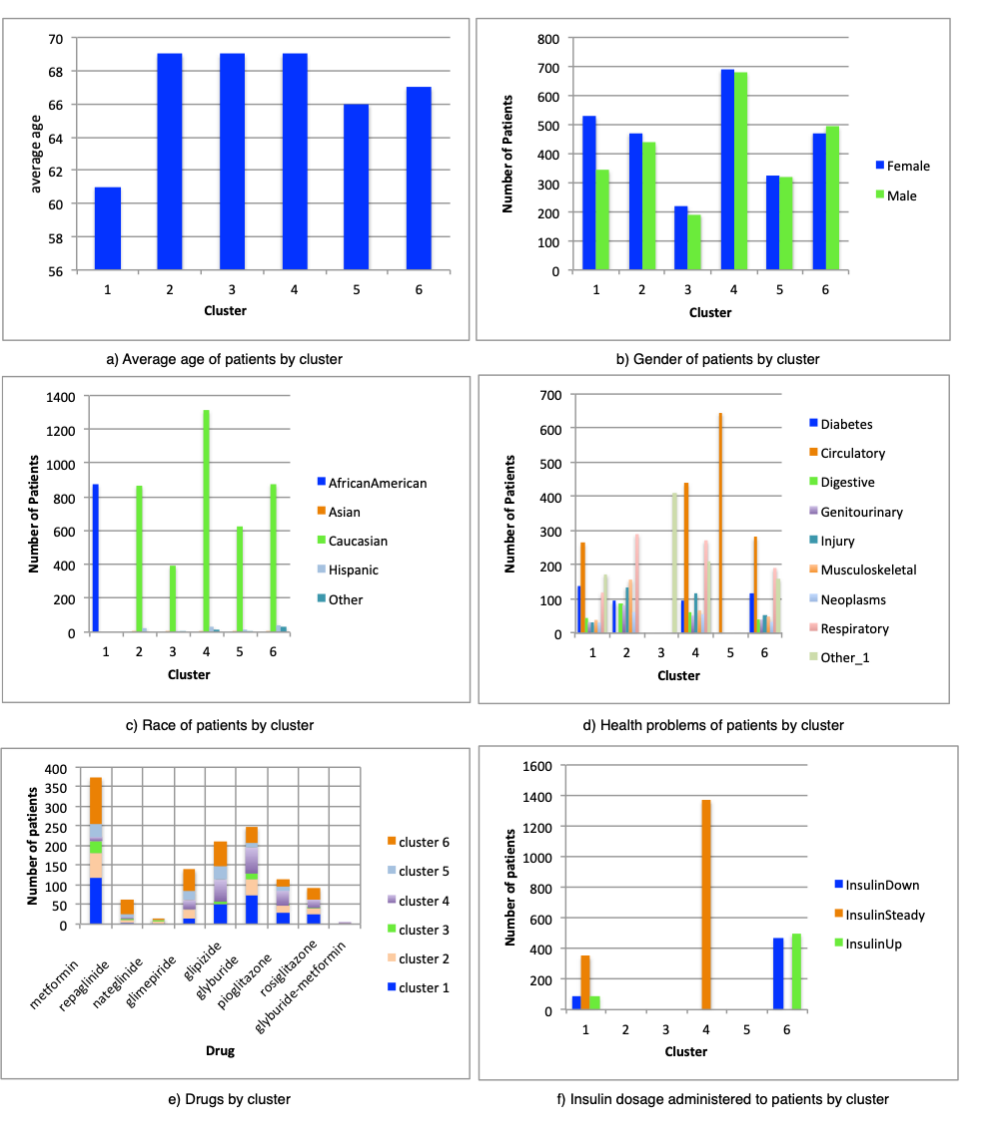
Details about the six clusters.

Figure 3 provides details about the six clusters, including the variables analyzed, such as age, gender, race, health problems, medications, and information about the doses of insulin administered to the patient.

**Table 5:** Comparative analysis of the performance of the algorithms.

Algorithm	Number of clusters	Silhouette coefficient	Execution time
K-means	6	0.654	15 minutes, 24 seconds
DBSCAN^a^	200	0.611	20 minutes, 31 seconds

^a^DBSCAN: density-based spatial clustering of applications with noise.

### Characteristics of Clusters

#### Cluster 1

Cluster 1 (n=875) included patients who were administered most of the drugs shown in Table 3. In this cluster, 60.6% (n=530) of the patients were female and the remaining 39.4% (n=345) were male. All patients in this group were of African American race. These patients suffered from health problems in the circulatory and respiratory systems directly related to diabetes. The most representative age range in this group was 45-75 years.

#### Cluster 2

Cluster 2 (n=910) was characterized by a homogeneous distribution of male and female patients; the main diseases diagnosed for these patients involved the respiratory system. Almost all of the patients in this group (n=867, 95.3%) were Caucasian. The main drugs administered for this group were metformin, glipizide, and glyburide, and the dosages were often changed (increased and decreased). Another relevant characteristic of this cluster is that the patients had not been administered insulin. The most representative age range of patients in this group was 55-85 years.

#### Cluster 3

In cluster 3 (n=412), the main drugs administered to patients were glyburide, metformin, and glipizide, whereas insulin had also not been administered to this group. There was diversity in the age range of the patients within this cluster, with the most representative range being 55-85 years. The patients in this cluster also tended to be diagnosed with other diseases such as metabolic disease and diseases of the nervous system. All of these patients belonged to the Caucasian race.

#### Cluster 4

The patients in cluster 4 (n=1369) were mainly administered insulin, metformin, and glipizide; although patients in this group were prescribed other drugs, their proportions were relatively lower. The main diseases diagnosed in this group of patients were diseases of the circulatory and respiratory systems. This cluster grouped the largest number of patients analyzed and the predominant age range was 55-85 years.

#### Cluster 5

Cluster 5 (n=646) was largely characterized by patients in this group having received more treatments with the following drugs: glyburide, glipizide, and metformin. Insulin had not been administered to this group, and all patients were diagnosed with diseases related to the circulatory system. The most prominent age range was 55-75 years.

#### Cluster 6

In cluster 6 (n=965), patients were mainly administered the following drugs: insulin, metformin, glipizide, and glyburide. The main diseases diagnosed in this group of patients were those of the circulatory system and diseases related to diabetes mellitus, among others. The predominant age range was 45-75 years.

### Quality of Predictions

To evaluate the quality of the predictions, we used the mean squared error (MSE), which penalizes more severely when the error is higher [20]. A significant MSE value of 0.53 was obtained in the test set, which means that the system is capable of obtaining good predictions. As a measure of error, a lower the error value indicates a more efficient RS [21].

### Quality of Recommendations

The quality metric is fundamental to measuring the performance of our RS, since it provides information on the proportion of recommended drugs that are relevant for the user. In our case, a drug is considered relevant when the value of the dose is greater than 1. In the experiments, a precision value of 0.61 was obtained, which indicated that the system provides acceptable recommendations.

### Generation of Recommendations

Considering a patient “p” with diabetes who belongs to cluster “c,” a list of drugs {f_1_,f_2_,...f_n_} with the highest prediction score is recommended by the system. An example of the recommendation for two patients in cluster 1 is presented in Table 6 based on a setting of providing the top 3 recommendations.

Table 6 shows that the recommended medications were similar for both patients in cluster 1; however, the order of recommendation varied according to the prediction score obtained for each medication. This is to be expected since these two patients share similar characteristics in terms of clinical information, personal data, and medical treatments stored in the data set.

**Table 6:** Drug recommendation for two patients with diabetes in cluster 1.

Patient ID	Drugs recommended^a^
36	Insulin, glipizide, metformin
15	Metformin, insulin, glyburide

^a^Drugs are listed in order of preference (highest to lowest prediction score).

## Discussion

### Principal Results

Our analysis of the UCI Machine Learning Repository showed that most patients with diabetes have circulatory and respiratory problems, followed by metabolic and nervous system problems. Regarding gender, women with diabetes showed more circulatory and respiratory health problems compared to men. It was determined that diabetes manifests differently for individual patients. In addition, in the analysis according to race (Caucasian, African American, Hispanic, and Asian), different categories were identified according to patient and clinical characteristics: (1) older patients with circulatory, respiratory, and other problems; (2) younger patients with digestive, respiratory, and other problems; (3) patients requiring increased insulin doses; and (4) patients prescribed more than one type of medication.

From the data set used, we further observed that diabetes can occur at any age; however, the disease appears to be more common among middle-aged and older people. The analysis showed that the health outcomes related to diabetes are different for each patient, both at the level of control with medication and health complications. Based on these findings, an RS is required to provide support to health care professionals to facilitate the management and control of this chronic disease. Therefore, a cluster-based RS was proposed to help recommend drugs to patients with diabetes. The clustering technique was used to identify groups of patients based on their similar characteristics, and the collaborative filtering technique was used to present information on the doses of the medications administered to patients. Our experimental results showed acceptable performance of the proposed system.

### Limitations and Future Work

The use of explicit information (ratings) from users enables making more precise recommendations; however, according to Wasid and Ali [22], additional effort is required from users when rating an item. Therefore, obtaining useful ratings without additional effort from users is one of the challenges of RSs based on collaborative filtering.

A typical problem of RSs based on collaborative filtering is that data and ratings are often scarce, resulting in a problem of new user and new item cold start. An alternative solution to overcome this problem is to calculate the similarity of the users based on user profiles [13]. For example, if two users are diagnosed with the same disease, they could be considered similar, even if they have not been administered the same drugs during their treatment. Other characteristics such as gender, age, medications administered to the patient, and diseases diagnosed could help classify patients into clusters. Although this study avoided the cold-start (new user) problem by using patient information for clustering and subsequent recommendation, there is a limitation with medications that are entered in the system and have not yet been prescribed to any patient, since the system would have difficulty recommending such medications (ie, new item cold-start problem).

One solution to this problem is to use the metadata of the new items when making recommendations [13]. Combining both user and item information would provide a hybrid approach to address the new user and new item cold-start problem. Therefore, as future work, we propose to (1) extend the recommendation approach using drug information for the prediction and recommendation process, and (2) consider the clustering results of the DBSCAN algorithm for prediction and analyze whether this can improve the quality of recommendations.

### Comparison With Prior Work

We found some previous studies related to the topic of RSs in the health domain; however, our proposed approach differs from these previous works by focusing on combining collaborative filtering with clustering techniques to avoid the cold-start (new user) problem.

The experimental results showed that our recommendation approach performs well in terms of offering good predictions and acceptable recommendations considering patient information. In comparison with the study of Sanchez et al [13] who recommended educational content about diabetes, our work focuses on recommending medications to patients with diabetes. Galiano and Paccanaro [23] used collaborative filtering for the prediction of medication side effects to provide recommendations to safety professionals based on a latent factor model. A latent factor method could also be considered for drug prediction to patients with diabetes, and these results can be compared with those obtained using the clustering-based recommendation approach. Consequently, RSs have been developed based on multiple methods that could be combined with clustering techniques to improve the recommendation process. For example, Chung and Jung [24] proposed a knowledge-based cluster model to improve prediction accuracy and make health care recommendations.

### Conclusions

We present an RS that is capable of suggesting medications suitable for patients with diabetes. This system considers user metadata to alleviate the cold-start (new user) problem, obtaining groups of patients with similar characteristics using clustering techniques, which are then used to recommend drugs for patients in the same cluster.

To measure the performance of the recommender system, the quality of the predictions and recommendations was evaluated. In the case of prediction accuracy, a significant MSE value was obtained and acceptable accuracy was found in the quality of recommendations, which can be further improved by using information from more drugs or combining with another collaborative filtering approach such as an item-based approach.

The proposed system offers a new method to provide support to health care personnel during the medical care of patients with diabetes by offering recommendations of possible medications that can be considered for the treatment of this disease. In addition, our RS has the advantage of providing recommendations that can be easily explained since the system recommends drugs that have been administered to patients with similar characteristics to the target patient. We believe that this system could be an important tool for health personnel, as it would help to streamline the process of health care and management.

## Abbreviations

DBSCAN: density-based spatial clustering of applications with noise
MSE: mean squared error
PCA: principal component analysis
RS: recommender system
UCI: University of California Irvine
